# BOV – a web-based BLAST output visualization tool

**DOI:** 10.1186/1471-2164-9-414

**Published:** 2008-09-15

**Authors:** Rajesh Gollapudi, Kashi Vishwanath Revanna, Chris Hemmerich, Sarah Schaack, Qunfeng Dong

**Affiliations:** 1 Center for Genomics and Bioinformatics, Indiana University, Bloomington, Indiana, USA; 2 Department of Biology, Indiana University, Bloomington, Indiana, USA

## Abstract

**Background:**

The BLAST program is one of the most widely used sequence similarity search tools for genomic research, even by those biologists lacking extensive bioinformatics training. As the availability of sequence data increases, more researchers are downloading the BLAST program for local installation and performing larger and more complex tasks, including batch queries. In order to manage and interpret the results of batch queries, a host of software packages have been developed to assist with data management and post-processing. Among these programs, there is almost a complete lack of visualization tools to provide graphic representation of complex BLAST pair-wise alignments. We have developed a web-based program, **B**LAST **O**utput **V**isualization Tool (BOV), that allows users to interactively visualize the matching regions of query and database hit sequences, thereby allowing the user to quickly and easily dissect complex matching patterns.

**Results:**

Users can upload the standard BLAST output in pair-wise alignment format as input to the web server (including batch queries generated installing and running the stand-alone BLAST program on a local server). The program extracts the alignment coordinates of matching regions between the query and the corresponding database hit sequence. The coordinates are used to plot each matching region as colored lines or trapezoids. Using the straightforward control panels throughout the web site, each plotted matching region can be easily explored in detail by, for example, highlighting the region of interest or examining the raw pair-wise sequence alignment. Tutorials are provided at the website to guide users step-by-step through the functional features of BOV.

**Conclusion:**

BOV provides a user-friendly web interface to visualize the standard BLAST output for investigating wide-ranging genomic problems, including single query and batch query datasets. In particular, this software is valuable to users interested in identifying regions of co-linearity, duplication, translocation, and inversion among sequences. A web server hosting BOV is accessible via  and the software is freely available for local installations.

## Background

The basic local alignment search tool (BLAST [1]), which allows for the comparison of similar sequences from the same species or across multiple species, has become one of the most popular bioinformatics programs used by biologists. This tool enables researchers to search their queries against sequence databases and produces an output of pair-wise alignments based on the query sequences and matching sequences from the database (referred to as hits). Although currently the majority of users utilize BLAST servers via the web which allow users to search against specialized databases (*e.g.*, NCBI [2], PlantGDB [3]), increasingly biologists are installing the BLAST program on their local computer in order to search against customized sequence collections (see tutorials for running locally-installed BLAST program, *e.g.*, [4]). As more sequence data have become accessible and the questions posed by genomicists have increased in complexity, additional programs have been developed to address some of the limitations of the basic BLAST capabilities, including those aimed at helping biologists post-process large BLAST outputs. Some examples include the MuSeqBox [5] and BioParser [6] programs, which can be used to flexibly select BLAST matching regions based on percent alignment coverage and identity, alignment scores, expectation value (E-value), and other attributes. In addition, the NuclearBLAST program [7] and the PLAN web server [8] allow users to store the plain BLAST text output in a relational database that enables advanced keyword searches for convenient data mining. Although the algorithms of such BLAST-utility programs are usually not sophisticated, their availability significantly improves the utility of the basic program and relieves the potential frustration of biologists who may otherwise be overwhelmed by having to manually analyze large sets of BLAST outputs.

In this same vein, we have developed a tool for the graphical output and visual analysis of matching regions between query and hit sequences identified by BLAST. This program is specifically designed to allow for the display of multiple regions of similarity between query and hit sequences which can be identified by BLAST, and has the additional benefit of handling single and batch query datasets. The typical BLAST output produces so-called High-scoring Segment Pairs (HSPs) that correspond to each matching region between the query and the database hit sequence. The query and hit coordinates of each HSP (*i.e.*, where the matching region starts and ends on the query and hit sequences) are embedded in the BLAST pair-wise alignment. In a hypothetical example, when a genomic region that contains both gene X and gene Y from species A is compared to the orthologous genomic region from species B that contains gene X' and gene Y' (orthologs of gene X and Y, respectively), the BLAST output may contain two HSPs that represent the matching regions between the two orthologous gene pairs. The distribution of multiple HSPs can be highly complex if gene duplications, inversions, and/or rearrangements have occurred in the regions being compared [9]. Even moderately complex multiple HSP distribution can be very difficult to interpret based on the raw BLAST pair-wise alignment output. Specifically, in order to find out which region of the query matches which region(s) of the hit sequence, the matching coordinates of each HSP must be mapped on both the query and hit sequences. Such a mapping process can be tedious if done by hand (*e.g.*, manually extracting each set of matching coordinates and drawing them on pieces of paper). To automate HSP mapping, we have developed a web program that parses the HSP coordinates of an uploaded BLAST output to generate interactive maps which graphically display matching regions. This tool can be used with typical single-query BLAST outputs obtained online or with batch query outputs generated by those users who have installed stand-alone BLAST programs locally on their computers.

## Implementation

**Figure 1:**
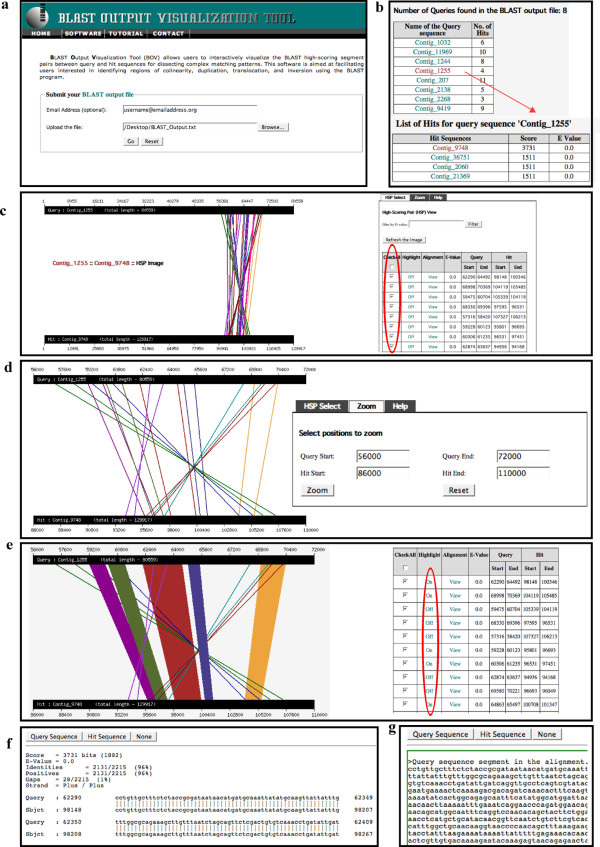
**Features of the BOV program**. Screenshots of the BOV program outlining key features of the application. (**A**) Main page showing input fields for the user's email and single or batch query BLAST output files. The user's email is requested in order to send a separate URL providing access to the BOV output file generated for any given input for 60 days. (**B**) A summary table of queries, including an individual link to the corresponding hits (indicated by the red arrow), is generated when batch query input files are uploaded. (**C**) All HSPs for a given query and hit sequences are initially displayed with colored lines connecting the start and end positions of each matching region along the sequence. Individual HSPs can be selected (indicated by the red circle) for in-depth analysis or the list of HSPs can be sorted or filtered to meet certain criteria prior to analysis. (**D**) The *Zoom *function can be used to examine a particular region along the hit or query sequence. (**E**) The *Highlight *function, activated by clicking on the column (indicated by the red-circle), changes the graphical display from lines (default) to trapezoids. (**F**) Pair-wise alignments for individual HSPs can be seen by clicking on the *Alignment *column for an individual HSP region. (**G**) The individual query or hit sequence region for each HSP can be viewed by clicking on the appropriate button at the top of the pair-wise alignment window.

As input, the BOV program takes a plain-text BLAST output file. As mentioned, the BLAST output can be generated from either single-query or multi-query DNA or protein BLAST searches (*i.e.*, BLASTN, BLASTX, BLASTN, TBLASTN, and TBLASTX). From the uploaded BLAST output file, the server will extract the length and description of each query and hit sequence, the coordinates of each HSP, the alignment score, and E-value using the freely available BioPerl package [10]. Specifically, the BOV program invokes the Bio::SearchIO module to convert plain-text BLAST output into BioPerl objects, which have built-in functions for extracting the information listed above. All the extracted information is temporarily stored in a simple MySQL relational database, which is designed to handle the many-to-many relationship between the query and hit sequences obtained from multi-query BLAST searches. Once the inputs are submitted, the user can immediately start to browse the resulting web pages, developed using Perl and DHTML programming language that interact with the BLAST data stored in the underlying database. For example, based on the E-value cutoff that users select, the underlying MySQL database is dynamically queried to retrieve the qualifying BLAST HSPs for display. Users are encouraged to follow the tutorial that is provided at the project web site to demonstrate the step-by-step usage of the BOV program (see features illustrated in fig. 1). If a multi-query BLAST output is submitted (fig. 1a), BOV organizes the information from each extracted query into a summary table so that it is convenient for users to choose their query sequence of interest for visual analysis (fig. 1b). Following the link under each query sequence, users will be guided to the core image display page, where they can interactively visualize the BLAST HSPs for any selected pair of query and hit sequences. The query and hit sequences are depicted as black horizontal bars. The default setting displays all the HSPs along the entire length of query and hit sequences. Each HSP is plotted as a pair of vertical or diagonal colored lines connecting the start and end positions of the corresponding matching region (fig. 1c, 1d). The standard Perl GD.pm library is used to draw the images (*e.g.*, lines and filled polygons). Through the control panel embedded in the image page, users can easily interact with the displayed HSPs. For example, the *HSP Select *function of the control panel allows users to choose a subset of HSPs for display by selecting/de-selecting the checkbox in front of each HSP (fig. 1c). Users can also filter the HSPs by E-value through a simple search form at the control panel. Selecting or filtering specific HSPs allows users to focus on particular regions of interest or on hits that meet certain criteria. In addition, users can apply the *Zoom *function to examine specific regions on either the query or hit sequences (fig. 1d). The default display option uses lines to indicate the boundary of each HSP in a given region (similar to the display style of SynBrowse [11]). If desired, a second display option is available and can be selected by clicking the *Highlight *link. In this format, a solid colored box similar to the 'trapezoid' display of SynView is used to show each matching region [12] (fig. 1e). Allowing users to control the display style of the image and allowing for the selection of one or multiple HSPs for visualization makes BOV a versatile tool for interpreting BLAST results. In addition, the images generated can be easily downloaded for user presentations or publications by following the embedded *Download Image *link. Although such graphic visualization is very convenient for exploring the matching topology of a region of interest, careful examination of the original BLAST output is also often essential. For example, manual inspection of the raw BLAST pair-wise alignments may be required to examine the quality of the HSPs (*e.g.*, whether there are any mismatches, insertions or deletions at critical active sites). For this purpose, each displayed HSP region is linked to the corresponding raw BLAST pair-wise alignment, so that users can inspect any particular region of interest in detail (fig. 1f). If a particular HSP appears to be part of an interesting matching pattern (*e.g.*, gene duplication or inversions), users can easily retrieve the actual sequence segment in the HSP by clicking the corresponding buttons embedded in the resulting alignment page (fig. 1g). The identity of the retrieved sequence segments can then be analyzed or investigated using other bioinformatics programs. Finally, when users submit their BLAST output, the server also requires the input of the user's email address. A URL link to a result summary page will be emailed to the user so that they can re-examine the same BOV output without submitting the input file again. The email also contains a link to access all the previously submitted jobs from the same user. For convenience, all the submitted files will be temporarily stored on our server for 60 days.

## Results and discussion

Visualization tools are critical for interpreting data and making discoveries in the area of bioinformatics, and especially in the field of comparative genomics. Often, complex changes in genome structure and organization (*e.g.*, gene duplications, inversions, and other rearrangements) are best identified, examined, and verified graphically rather than via the automated numerical or textual output of most computer programs. For whole-genome comparisons, sophisticated tools such as VISTA [13], GenAlyzer [14], SynBrowse [11], Sybil [15], Cinteny [16], and AutoGRAPH [17] have been developed and are popular within the bioinformatics community. Many biologists, however, may find it inconvenient to either install the software (*e.g.*, SynBrowse and its prerequisites can present a serious challenge to install and update, even for bioinformaticians), or to prepare required data input files (*e.g.*, the specific GFF-format files required by SynBrowse may require computer programming). On the other hand, many biologists have already become adept at using the well-known BLAST program and have applied it to a diverse number of research questions. In fact, many comparative genomics projects are first initiated by a simple BLAST search. Despite the popularity of the BLAST program, there is no published visualization tool that converts the raw BLAST pair-wise alignment into a straightforward display plotting the positions of multiple HSPs. Such a visualization tool can allow biologists to easily and quickly investigate gene or genome structures in a comparative genomics context, without the potential hassles of having to invoke the heavyweight genome browsers mentioned above.

Although few visualization programs directly related to BLAST outputs are currently available, some do exist that attempt to satisfy the most common goals in comparative genomics research. For example, Durand *et al*. [18] published the Visual BLAST program. Although its accompanying paper describes several useful features for analyzing BLAST outputs, the computer program was designed to run using the now obsolete Microsoft Windows 95/NT operating system and the web site hosting the program is no longer available. In addition, based on the description of their published paper, Visual BLAST does not provide the plotting function for HSPs available in BOV. Another program, BLAST2GENE [9], converts BLAST output into a graphical plot (although very different than the ones produced by BOV). However, the BLAST2GENE program is only designed to compare one small gene sequence against a larger genomic region in order to identify all the similar gene copies in the latter. As a result, only an asymmetric diagram is plotted to indicate the HSP positions on the larger genomic region. In addition, its accompanying web server can only handle single-query BLAST outputs. In contrast, the BOV program treats both the query and hit sequence equivalently and both can be long sequences. In addition, the BOV server can process multi-query BLAST outputs and can produce a useful graphic for publication and presentation (easily available for download).

Originally, the development of BOV was prompted by our need to carefully analyze the BLAST comparisons among a set of *Daphnia pulex *genomic contigs (for an example of the application of BOV using this output, see fig. 1). After submitting our BLAST output to the server, a large-scale overview of the BLAST alignment regions between the assembled *Daphnia *genomic contig #1255 (total length 80,559 bp) and genomic contig #9748 (total length 129,917 bp) are produced (fig. 1c). Although the two contigs are quite long, the matching regions are limited to near the 3' portion of both contigs (fig. 1c). After zooming into the high-score matching region, some complicated matching patterns can be identified (fig. 1d, 1e). Five blocks of genomics regions show perfect co-linearity between these two contigs (colored trapezoids in fig. 1e, the query regions 59228–60123 in dark magenta, 60306–61235 in dark olive green, 62290–64492 in brown, and 64863–65497 in dark slate blue); five blocks of regions of contig #1255 show inversion and/or translocation in the corresponding regions of contig #9748 (colored lines in fig. 1e, query regions 57316–58420 in dark green, 59475–60704 in dark blue, 68330–69396 in dark cyan, 62874–63637 in dark orchid, and 69580–70221 in dark red). In addition, several regions of contig #9748 are duplicated in contig #1255 (*e.g.*, the hit region 104119–105485 matches to both the query region 59475–60704 and 68998–70369, with the inversion of the query region 59475–60704). The plot of the matching regions (fig. 1e) makes it much more straightforward to fully understand the topology of complex genomic regions, including stretches of co-linearity, inversion, translocation, and duplication. In addition, using the tools embedded in BOV, it is possible to retrieve the sequence segments from the above matching regions for further study of their identities. Out of the ten highlighted matching regions, four match to *Daphnia *EST sequences (*e.g.*, the query region 62290–64492 in brown; data not shown) indicating that those HSP segments correspond to actively transcribed genic regions. Using BLAST to search for matches among these regions and the NCBI *nr *and *nt *databases, we find no hits to known genes or repetitive elements (data not shown) indicating that, although there may be functional significance to at least some of these regions, the identification of candidate homologous genes or functional domains will require further investigation.

We envision that BOV will be very useful for biologists interested in examining the evolution of gene structures (including intron/exon turnover across species), relationships among orthologous and paralogous genes, analysis of repetitive elements or tandem arrays, as well as the identification of regions of small or large scale synteny along chromosomes (including inversions, translocations, and gene duplications). The BOV tool provides a freely accessible web server for biologists with no software installation or maintenance required by the user. Users can simply upload their BLAST output and follow the intuitive web interface to visualize the mapping of the HSPs. Similar to other BLAST-utility programs, BOV is not sophisticated in its computer science algorithms and can be downloaded in its entirety for use and/or modification depending on specific analytical needs. For example, although the BOV program is designed to process BLAST outputs, the parsing component can be easily replaced to handle any other pair-wise alignment program using our drawing routines. We believe that the availability of BOV enriches a biologist's tool kit for effectively processing BLAST output and conducting comparative genomic research.

## Conclusion

The BLAST program is widely used in genetic and genomic research. We have developed a web server, BOV, to provide a visualization tool for biologists to conveniently dissect the BLAST output for complex matching patterns. Our program allows for single- or batch-query manipulation, can be easily accessed and downloaded for use and modification, and provides a user-friendly web interface to interactively visualize the matching regions of query and database hit sequences.

## Availability and requirements

The BOV program is freely accessible, using a web browser at . The software is also available from the web site for local installation. We have made BOV portable across Linux and UNIX distributions, and compatible with BioPerl 1.4 (or higher version) and MySQL 5.0 (or higher version). We have tested the BOV installation on SunOS 5.11(i386), Ubuntu Linux server v2.6.24, and Gentoo Linux x86_64 v2.6.23. BOV can be viewed with FireFox 1.5, Opera 9.27, Safari 3.0, Internet Explorer 7.0, or their higher version. The BOV website will be updated to contain the latest information on operating system and software compatibility.

**Project name**: BOV

**Project home page**: 

**Operating systems**: Local installation requires Linux/UNIX.

**Programming language**: Perl, JavaScript

**License**: The software is under the Apache license 2.0.

## Authors' contributions

RG and KR implemented the computer program and web server. CH guided all software engineering aspects of the system for quality management. SS tested the system and provided on-line descriptions of the tool, and critically revised the manuscript. QD designed and supervised the project, and prepared the manuscript. All authors read and approved the final manuscript.
